# Alkynes as Synthetic Equivalents of Ketones and Aldehydes: A Hidden Entry into Carbonyl Chemistry

**DOI:** 10.3390/molecules24061036

**Published:** 2019-03-15

**Authors:** Igor V. Alabugin, Edgar Gonzalez-Rodriguez, Rahul Kisan Kawade, Aleksandr A. Stepanov, Sergei F. Vasilevsky

**Affiliations:** 1 Department of Chemistry and Biochemistry, Florida State University, Tallahassee, FL 32306, USA; egonzalez@chem.fsu.edu (E.G.-R.); kawaderahul84@gmail.com (R.K.K.); 2 Institute of Chemical Kinetics and Combustion, Siberian Branch of the Russian Academy of Science, 630090 Novosibirsk, Russia; stepanov@kinetics.nsc.ru (A.A.S.); vasilev@kinetics.nsc.ru (S.F.V.); 3 Novosibirsk State University, 2, Pirogova Str., 630090 Novosibirsk, Russia

**Keywords:** alkynes, carbonyl compounds, ketones, aldehydes, condensations, cyclizations, catalysis, nucleophilic addition, acetals, rearrangements, electronic structure

## Abstract

The high energy packed in alkyne functional group makes alkyne reactions highly thermodynamically favorable and generally irreversible. Furthermore, the presence of two orthogonal π-bonds that can be manipulated separately enables flexible synthetic cascades stemming from alkynes. Behind these “obvious” traits, there are other more subtle, often concealed aspects of this functional group’s appeal. This review is focused on yet another interesting but underappreciated alkyne feature: the fact that the CC alkyne unit has the same oxidation state as the -CH2C(O)- unit of a typical carbonyl compound. Thus, “classic carbonyl chemistry” can be accessed through alkynes, and new transformations can be engineered by unmasking the hidden carbonyl nature of alkynes. The goal of this review is to illustrate the advantages of using alkynes as an entry point to carbonyl reactions while highlighting reports from the literature where, sometimes without full appreciation, the concept of using alkynes as a hidden entry into carbonyl chemistry has been applied.

## 1. Introduction

Alkynes, one of the most familiar “textbook” functionalities, are highly versatile and useful, as illustrated by their widespread application in different fields of chemistry, biology and materials science [1,2]. However, despite being familiar, alkynes display a number of interesting and underappreciated electronic features. For example, alkynes’ high energy [3] confers them an edge by making alkyne synthetic transformations highly thermodynamically favorable, and generally irreversible. Likewise, the presence of two orthogonal π-bonds that can be manipulated separately supports flexible synthetic strategies that render alkynes valuable for the design of efficient cascade transformations [4]. Furthermore, hidden behind these “obvious” traits are other more subtle aspects of this functional group’s appeal. For example, alkynes can be considered as super-stabilized 1,2-dicarbenes [4,5], as well as a perfect atom-economical, carbon-rich building unit for the preparation of extended polyaromatics [3,6,7,8,9,10,11,12,13,14,15,16,17,18,19,20,21,22,23].

**Scheme 1:**
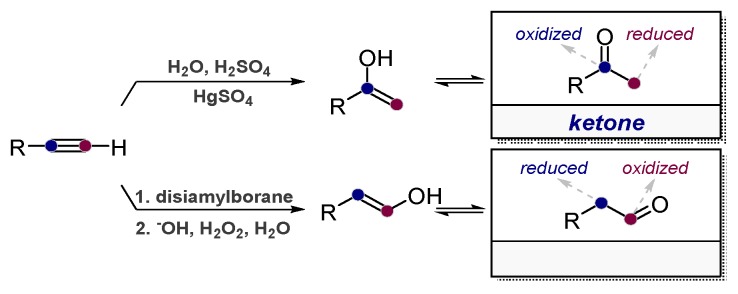
Markovnikov (top) and anti-Markovnikov (bottom) hydration of alkynes converts them into either ketones or aldehydes, respectively.

This review will focus on yet another interesting alkyne feature, i.e., the fact that the -C≡C- alkyne unit has the same oxidation state as the -CH_2_C(O)- unit of a typical carbonyl compound. This equivalency is readily illustrated by the fact that alkynes are converted to carbonyl compounds via hydration (Scheme 1). Although in this process one of the alkyne carbons is formally oxidized while the other one is formally reduced, hydration is obviously not a redox process, and water is neither an oxidant nor a reductant.

Thus, “classic carbonyl chemistry” can be accessed through alkynes, and new alkyne cascade transformations can be engineered by unmasking the hidden carbonyl nature of alkynes. The goal of this review is to illustrate the advantages of using alkynes as an entry point to carbonyl reactions.

The connection between alkynes and carbonyl compounds is generally mediated by formation of bonds between alkyne carbons and heteroatoms. In a similar fashion to water addition to alkynes, additions of other nucleophiles do not change the overall oxidation state of the functional group. For example, the addition of O- or *N*-centered nucleophiles transforms an alkyne into enol and enamine derivatives respectively. Although the addition of *C*-centered nucleophiles to alkynes is an overall reduction from a formal point of view, it can also give access to carbonyl derivatives if the intermediate vinyl anion is trapped by a reaction with a heteroatomic electrophile or an oxidant [24,25]. Alternatively, the “preoxidized” alkynes (ynols [26] or ynamine derivatives [27]) can enter the carbonyl reaction part of the reaction hypersurface after reaction with a C-nucleophile directly, without a need for an additional oxidant. It is worth noting that reactions of alkynes with heteroatomic *electrophiles* lead to products with higher oxidation states (e.g., α-dicarbonyls) but we will not discuss such transformations in the present review.

**Scheme 2:**
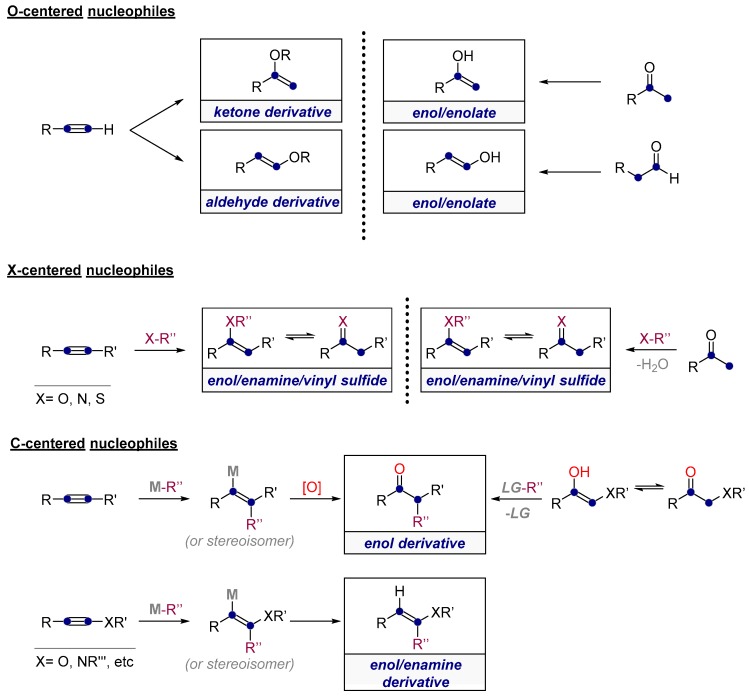
Synthetic equivalency of alkynes and carbonyl compounds.

Given that the same intermediates (e.g., enols and enamines) can be accessed starting from a carbonyl compound, the alkyne and the carbonyl functionalities are conceptually equivalent as two possible entry points to the many useful processes where such reactive intermediates are involved (Scheme 2). From this, one can visualize how alkynes and carbonyls lie on the same potential energy surface as the two energy minima connected via a multitude of routes –including those that traverse enols and enamines.

However, of course, there are differences between the two functionalities as well, and this is where it gets interesting. Due to these differences, each starting material (alkyne vs. carbonyl) possess their own advantages and disadvantages. Let’s discuss them from a conceptual perspective.

### 1.1. The High Energy of Alkynes Can be Used to Drive Difficult Transformations

**Scheme 3:**
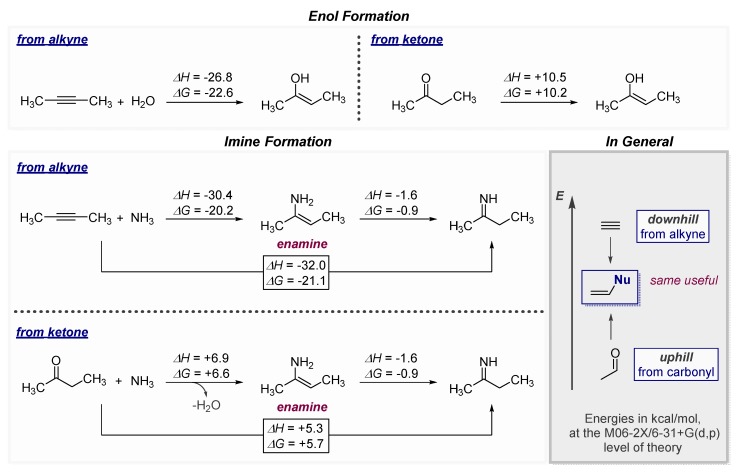
Thermodynamics of enol and enamine formation from an alkyne and a ketone.

In general terms, the high energy stored in alkynes [3] can be a strong advantage in reaction design. To illustrate the vastly different amount of energy stored in alkynes relative to carbonyls, it is instructive to compare the thermodynamic parameters for the transformation of an alkyne and a carbonyl compound to their corresponding enol (Scheme 3). Whereas the reactions of the model alkyne (2-butyne) are highly favorable (~−20 kcal/mol!), the analogous reactions of the model ketone (2-butanone) are 6–10 kcal/mol uphill. These differences clearly show the energetic advantage of accessing enols and enamines from alkynes. No thermodynamic penalty needs to be paid in such transformations.

### 1.2. Low Polarization of Alkynes Endows Them with Flexible Selectivity

**Scheme 4:**
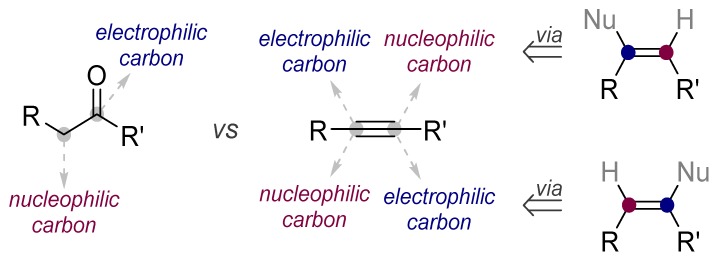
Each alkyne opens not one but two doors into carbonyl chemistry.

Another key feature that gives alkynes more versatility than their carbonyl equivalents arise from the fact that a non-symmetric alkyne can yield two distinct carbonyl compounds. For example, terminal alkynes can be synthetic equivalents of either ketones or aldehydes depending on the “Markovnikov vs. anti-Markovnikov” [28,29] regiochemistry of their addition reactions. It is through this “divergent selectivity” that the alkyne, like Schrodinger’s cat, displays its intrinsic duality—each of the alkyne carbons has the potential to be either oxidized or reduced in the process of nucleophilic addition. It is the nucleophile that chooses between the two potentialities. In short, each alkyne opens not one but two doors into carbonyl chemistry (Scheme 4).

Of course, the lack of polarization has a flip side as well. Because the carbonyl group is highly polar, aldehydes and ketones are “pre-activated” for reaction with nucleophiles and their selectivity is “pre-programmed”. The alkyne moiety is less polarized and generally needs an additional activation step to interact with a nucleophile. Although the need for preactivation may look like a *disadvantage*, it also offers *an opportunity* for the design of catalytic processes with potential advantages for the control of selectivity.

**Scheme 5:**
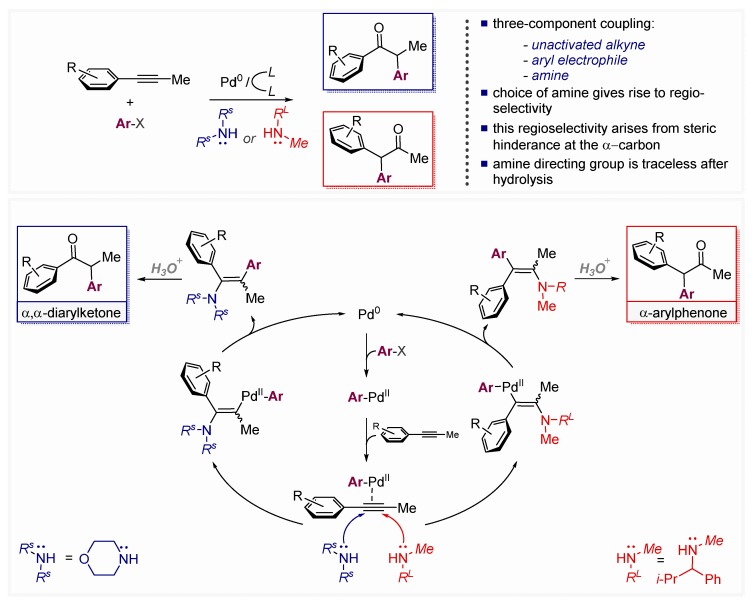
Synthesis of α-arylphenones and α,α-diarylketones through directed catalytic alkyne arylations.

Let us illustrate the divergent selectivity that can arise from regioselective control of nucleophilic addition to alkynes by using an elegant example reported by Park and coworkers [30]. This work discloses an elegant way to control the regiochemical outcome of alkyne derivatization. Here, the use of amines as directing groups facilitated the palladium-catalyzed arylation of alkynes and, through the choice of substituents on the amine, dictated which of the two regioisomeric aminopalladation intermediates is preferred. The final reductive elimination step completes the regioselective alkyne aminoarylation sequence. Finally, hydrolysis of the resulting enamine yields either the α-arylphenone or α,α-diarylketone, depending on the amine choice (Scheme 5).

This example clearly highlights that an alkyne starting material has the potential to give two distinct isomers as products, and that the selectivity can be tuned on demand to provide either of the two products. Such diverging one-step reactivity would be impossible from a single carbonyl precursor.

In the following sections, we will show other approaches to the control of regioselectivity for nucleophilic additions to alkynes. For example, the use of alkynes as cyclization precursors also gives an opportunity to control the regioselectivity of addition by using the stereoelectronic preferences for cycle formation [31]. Sometimes, these preferences can even override important factors such as alkyne polarization as will be illustrated by the “anti-Michael” additions provided in Section 3.2.

## 2. Alkynes in Ketal Formation

In addition to the high energy content and the potential for tunable divergent selectivity, the use of alkynes as carbonyl surrogates has other advantages. For example, the alkyne functionality is relatively kinetically inert (i.e., the alkyne π-bonds are strong) and, in the absence of strongly donor or acceptor substituents, has no intrinsic polarity. The chemical inertness of alkynes under various reaction conditions accounts for their broad tolerance in transformations that would be problematic for other functionalities, such as carbonyls. Of course, chemical inertness can be both a blessing and a curse but, in the case of alkynes, a breakthrough that greatly facilitated their application in chemical synthesis came with the development of alkyne-selective (“alkynophilic”) π-acidic metal catalysts [32,33,34,35,36,37]. Such selective catalysts (especially, the cationic Au-species) allowed chemists to “unlock” alkyne reactivity on demand with a large degree of control. To illustrate this advantage, let’s take the textbook reactions of *O*-nucleophiles with carbonyl groups. These reactions have found widespread applications ranging from a simple protecting group installation to the late stage functionalization in the preparation of complex molecular architectures. One of the problems inherent to this approach is that the chemo- and regioselective reactions at the carbonyl moiety can be difficult in the presence of several carbonyls [38]. The ability to use an alkyne selectively as a carbonyl equivalent even in the presence of carbonyl groups allows for selective functional group manipulations. Moreover, the use of non-carbonyl precursors, e.g., alkynes, can provide elegant synthetic strategies where the alkyne moiety serves as a protecting group for a carbonyl. Because the alkynes are less polar and inherently less electrophilic than carbonyls, their use expands the range of possible reaction conditions and provides additional flexibility in the reaction design.

**Scheme 6:**
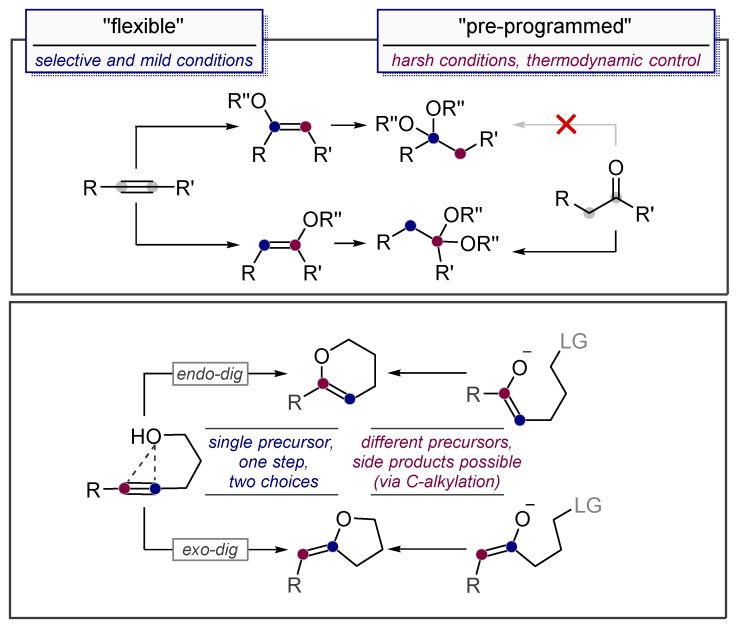
Comparison of alkynes and carbonyls as precursors for vinyl ethers.

Comparison of alkynes and carbonyls as starting materials for the synthesis of enol ethers and ketals is given in Scheme 6. In the absence of structural restraints (e.g., a non-enolizable part of the molecule), a typical carbonyl precursor can form two enolates (endo- and exo-isomers), each of which can undergo an exo-tet-cyclization by displacing a leaving group at a suitable position. The selectivity here is controlled by the relative abundance of the two enolates and their relative reactivity in the respective cyclization steps. Although such processes can be controlled and used efficiently, such control is not always trivial. On the other hand, homogeneous Au-catalysis gives direct access to vinyl ethers through hydroalkoxylation of alkyne/catalyst complex [39,40,41]. The process is catalytic, atom-economical and scalable.

**Scheme 7:**
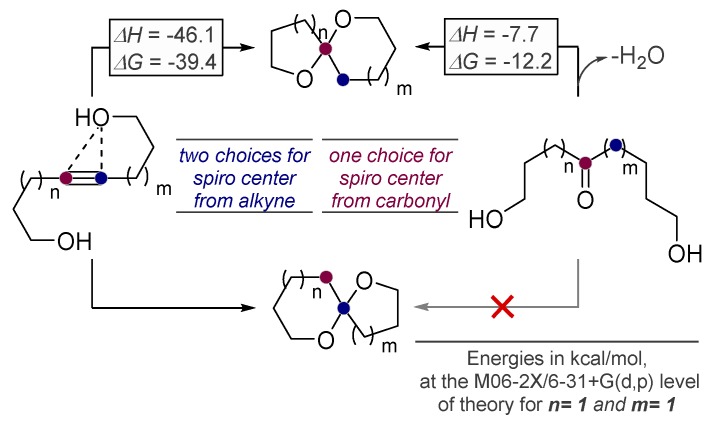
Comparison of alkynes and ketones in intramolecular spiro-ketalizations.

In the presence of suitable π-acidic catalysts, the vinyl ethers obtained from alkynes can also be further converted into ketals. This powerful technique provides an atom economical and redox neutral approach in late stage spiroketalization. Although intramolecular ketalization of carbonyls is more favorable thermodynamically than its intermolecular version (Scheme 7 vs. Scheme 3), use of alkynes comes with additional synthetic flexibility as illustrated in Scheme 7.

**Scheme 8:**
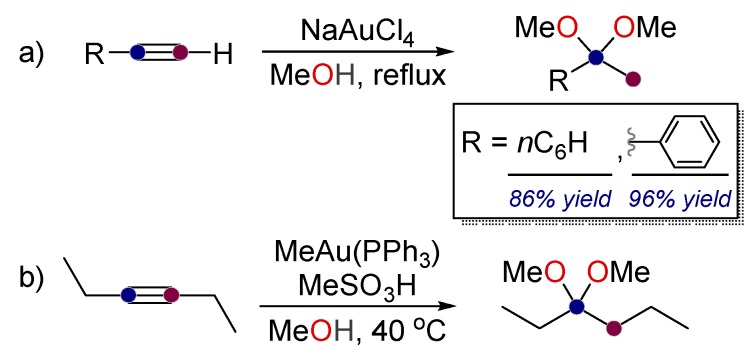
Utimoto’s Au-catalized acetal formation from alkyne starting materials.

Both inter- and intramolecular alkoxylations of alkynes have been reported using various alkynophilic metal salts. In 1991, Utimoto [42] and coworkers showed that alkynes can be directly converted into acetals or ketals using NaAuCl_4_. They have successfully converted terminal acetylenes into dimethyl acetals in excellent yields in refluxing methanol (Scheme 8a). However, Au (III) is quickly reduced into the inactive metallic gold. Shortly thereafter, Teles [43] and coworkers found that a new class of cationic [L-Au]^+^ complexes (where L is phosphine, phosphite or arsine) serve as excellent catalysts for the addition of alcohols to alkynes (Scheme 8b). These catalysts achieve total turnover numbers of up to 10^5^ moles of product per mole of catalyst, and they are neither water nor air sensitive. However, the reaction has a drawback, as it uses concentrated solutions of strong acids.

**Scheme 9:**
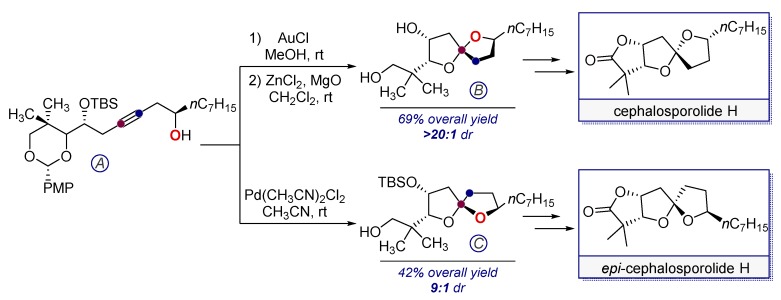
Dudley’s intramolecular bishydroxylation of alkynes.

The advantages discussed above are displayed in many examples, including those from the Trost [44], Furstner [45], Dudley [46], and Forsyth groups [47]. For example, in 2010, Dudley and coworkers reported the synthesis of cephalosporolide H, an anti-inflammatory agent containing a 5,5 spiroketal system [46]. A key spiroketal-forming system step in the synthetic route towards this natural product was accomplished through an Au(I) catalyzed hydroxylation of an alkyne precursor (Scheme 9, top).

This report clearly illustrates the advantage of the alkyne functionality over the carbonyl group, as alkyne activation is achieved through the selection of suitable π-acidic metal catalysts and further fine-tuned by choosing the right ligands to complete the coordination sphere of the catalytic metal. For example, the Au(I) catalyzed cyclization of alkyne A in MeOH gives rise to a 1:1 mixture of epimeric 5,5 spiroketals B and C. This mixture can be isomerized into a single diastereomer (in 86% yield) with ZnCl_2_, a Lewis acid that is capable of chelation with the pendant hydroxy group and the spiroketal oxygen of the adjoining ring. Alternatively, alkyne A can be converted selectively into the epimeric 5,5-spiroketal C by using Pd(CH_3_CN)_2_Cl_2_ as the catalyst.

**Scheme 10:**
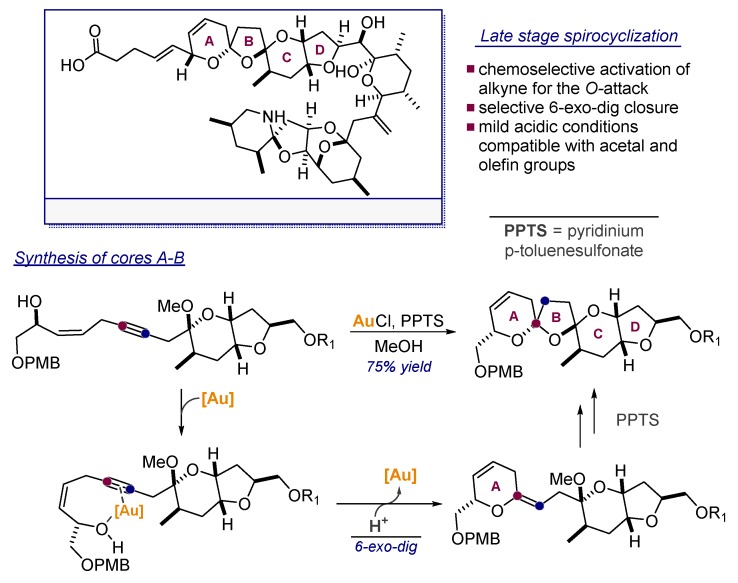
Gold catalyzed bis-alkoxylation of alkynes in the synthesis of spiroketals by Forsyth and coworkers.

Similarly, Forsyth and coworkers recognised that highly oxygenated spiro-ring systems of azaspiracid marine toxins can be accessed through bis-hydroalkoxylation of an alkyne precursor (Scheme 10).

The oxadispiroketal A-D ring system of this natural product was assembled by Au-catalyzed 6-exo-dig cyclization of an internal alcohol onto the alkyne [47]. Ring size and strain of the product determine the regioselectivity of the initial attack of the hydroxy group on the alkyne–Au (I) complex. The resulting enol ether product consequently undergoes PPTS catalyzed spiro B-ring formation in a 75% yield under thermodynamically controlled reaction conditions. The authors also suggest that the use of an alkyne as a surrogate for a ketone reduces the possibility of alkene isomerization.

## 3. Vinyl Ether Generation from Alkynes

In this section, we will show how the connection between alkynes and enol derivatives can be exploited for the preparation of heterocycles. From a practical point of view, the use of carbonyl vs. alkyne precursors as starting materials for a one-step synthesis of vinyl ethers is complementary.

**Scheme 11:**
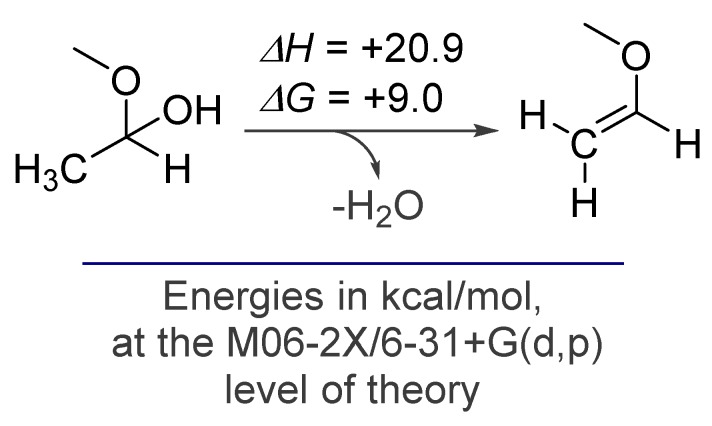
Thermodynamics of hemiacetal/vinyl ether transformation can be unfavorable.

It is important to note at this point that carbonyls are, of course, versatile synthetic precursors as well. For example, although carbonyls are intrinsically electrophilic, they can be converted into nucleophiles via enolization. In their parent electrophilic form, carbonyls can react with O-nucleophiles with the formation of hemiacetals, a process that, as any sugar chemist can testify, is quite favorable in its intramolecular version. The hemiacetals can be, in principle, converted into vinyl ethers but this process is not always thermodynamically favorable (Scheme 11).

Alternatively, cyclic vinyl ethers can be formed directly when nucleophilic carbonyl-derived functionalities (i.e., enols or enolates) attack an electrophilic carbon with a suitable leaving group. Such processes are well-known but, as we will discuss below, they are quite complex from the stereoelectronic perspective.

When alkynes are used as the starting material, they are generally activated towards the intramolecular nucleophilic attack (often as a π-complex with a suitable Lewis acid). As we will show below, this activation is important from both the kinetic (decreasing the addition barrier) and thermodynamic (stabilizing the cyclization product) points of view. Furthermore, such coordination can facilitate synthetic access to the less favorable “endo-dig” version of alkyne cyclizations via the so-called “LUMO Umpolung” (vide infra) [48,49].

**Scheme 12:**
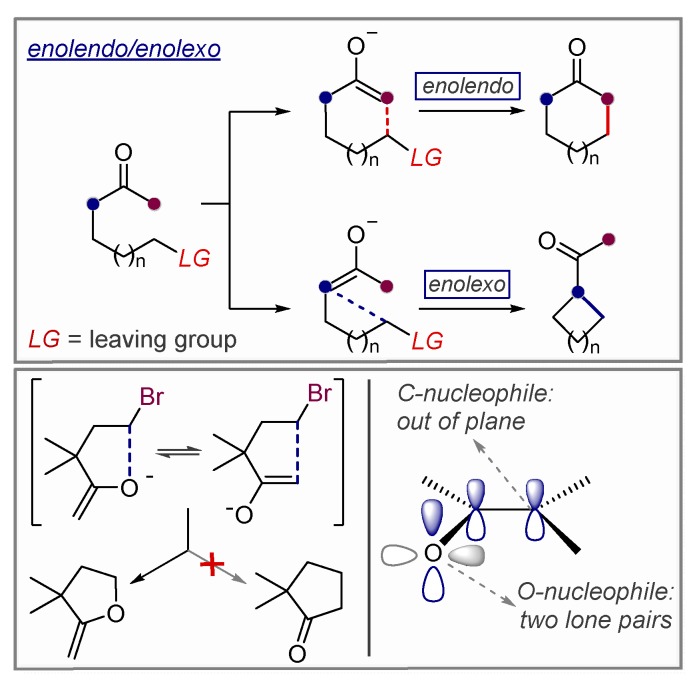
Top: Two patterns (C-enolexo and C-enolendo) for exo-tet cyclizations. Bottom: Although cyclizations of enolates can occur at either the carbon or oxygen, this process is controlled by stereoelectronic factors (see discussion in text).

Rules for enolate cyclizations are relatively complex because *two* orbital arrays need to be aligned in the cycle-closing bond forming step [50,51]. The enolate C=C bond can be exocyclic to the newly formed ring in the so-called “enolexo” closures or, alternatively, the enolate can be endocyclic to the new ring in the “enolendo” closures. Furthermore, the enolate closure can occur at either the carbon or oxygen (Scheme 12). Baldwin’s rules for enolates suggested that 3- and 4-(enolendo)- closures for both exo-tet and exo-trig modes are unfavorable cyclization modes, while 5- and 6-(enolendo)- and 3- to 7-(enolexo)- were classified as favorable. Even now, not all cyclization modes for the enolates have been systematically explored.

**Scheme 13:**
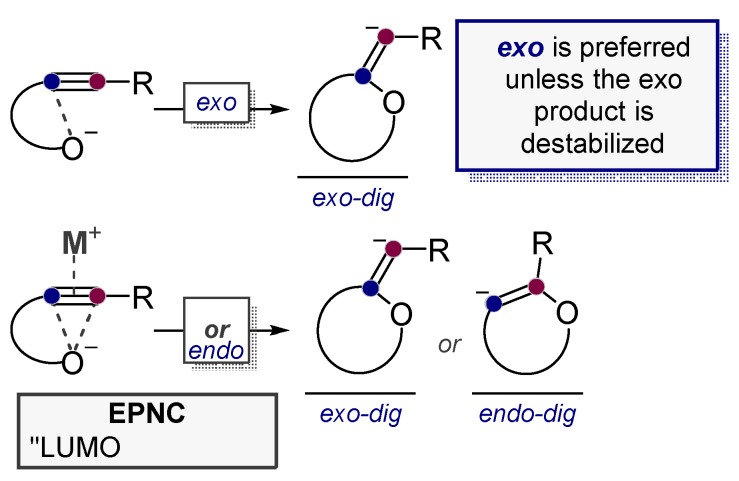
Approaches to selective exo-dig and endo-dig cyclizations can be accomplished by using either a classic anionic or a Lewis acid-mediated (the so-called “Electrophile-Promoted Nucleophilic Closure (EPNC) processes) pathways with different stereoelectronic requirements.

Rules for alkyne cyclizations (often referred to as “the dig-cyclizations”) are less complex but more tunable. For anionic nucleophiles, exo-cyclizations are preferred in the absence of large thermodynamic or electronic bias [52]. This preference originates from the stereoelectronic preferences for nucleophilic attack at the alkyne π*-orbital. Coordination of the alkyne to a Lewis acid leads to the removal of the orbital symmetry restrictions (via “LUMO Umpolung” [48], Scheme 13) and allows for both exo-dig and endo-dig processes to proceed without stereolectronic penalty.

### 3.1. Anionic Cyclizations without Alkyne Preactivation

**Scheme 14:**
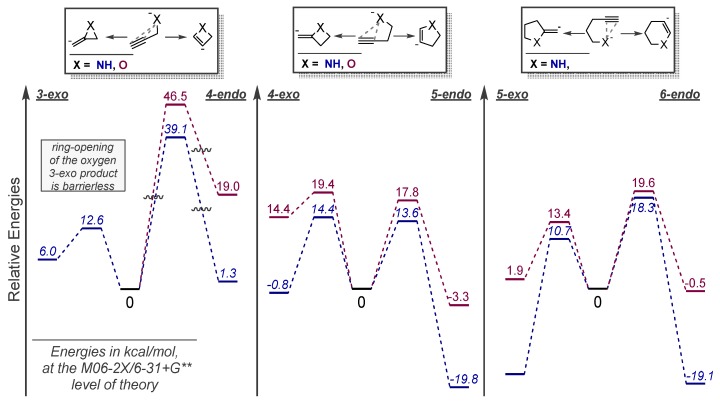
Potential energy surfaces for selected exo-dig and endo-dig anionic cyclizations of *N*- (blue dashed, *italic*) and *O*- (violet dashed, non-italics) anions with terminal alkynes.

For the intramolecular attack of negatively charged nucleophiles at alkynes, the general stereoelectronic factors favor exo cyclizations [52]. Under kinetic control, the exo preference is clear when the two possible cyclic products have rings of similar size and strain (i.e., either both are strained for the 3-exo/4-endo pair or both are relatively strain-free -the 5-exo/6-endo pair). A peculiar exception is the case of 4-exo and 5-endo-dig anionic closures where the two barriers are quite similar (Scheme 14, middle) [53]. This seemingly irregular trend originates from the interplay of stereoelectronic factors with thermodynamic contributions to the activation barrier [54,55]. Because the exo-product is much more strained than the endo-product in the 4-exo/5-endo pair, the endo-cyclization is much more exothermic and the exo/endo kinetic competition becomes relatively close (Scheme 14).

Note, however, that cyclizations of the *N*- and *O*-centered anions onto an alkyne transform a *hetero*atom-centered anion into a *carb*anion. The energy cost due to this unfavorable change is substantial. As the result, formation of small cycles, especially from alkoxide anions is often endergonic and reversible (Scheme 14). Even for the kinetically favorable 5-exo/6-endo-dig cyclizations, the formation of these relatively large cycles formed from the parent anionic oxygen systems is close to being thermoneutral. We will show in one of the following sections how this feature can be used for introduction of elements of thermodynamic control in these cyclizations.

### 3.2. Use of Stereoelectronic Exo-Preference for Overriding Alkyne Polarizations

**Scheme 15:**
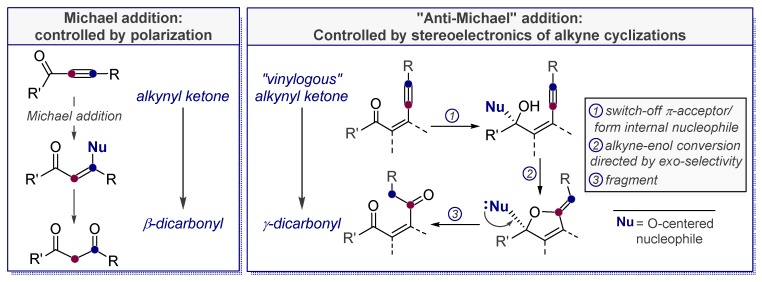
Two approaches to regioselective alkyne/carbonyl transformations.

In this section, we will illustrate some of the features for alkyne cyclizations, mostly using *O*-nucleophiles. The key preference is that for the exo-cyclizations. Sometimes, this stereoelectronic preference is strong enough to cause additions that go against alkyne polarization such as the “anti-Michael” intramolecular additions to alkynyl ketones (Scheme 15).

**Scheme 16:**
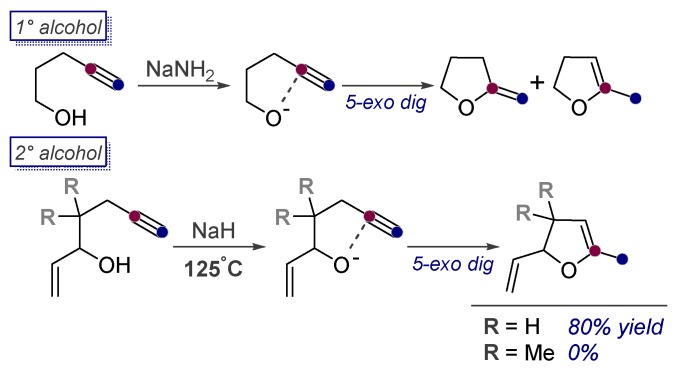
Base promoted 5-exo-dig cyclizations of primary and secondary alcohols onto terminal triple bonds.

Base-catalyzed cyclizations of aliphatic alcohols exclusively follow the 5-exo-path where the cyclic vinyl anion is trapped by protonation, thus activating another acyclic alcohol. When a secondary allylic alcohol [56] is used as opposed to a primary alcohol [57,58], the process becomes more sluggish and *gem*-dimethyl groups are required (Scheme 16). Addition of an anion-stabilizing group at the terminal carbon renders 5-exo-dig cyclizations of oxygen nucleophiles more efficient [59,60].

**Scheme 17:**
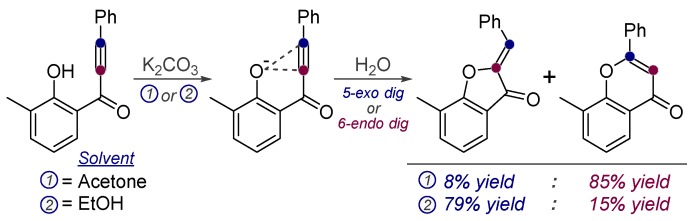
The solvent dependent cyclizations of oxygen anions with three sp^2^-atoms in the linking chain.

In those cases, where the exo- and endo-cyclizations are intrinsically close, reaction conditions, such as the choice of solvent can be used to control regioselectivity. For example, Miranda and coworkers [61] found that the “anti-Michael” exo-dig product is formed (79% yield) when the cyclization is carried out in a protic solvent like ethanol (Scheme 17). In this interesting example, the stereoelectronics of cyclization go against alkyne polarization in determining the regioselectivity of alkyne reaction. On the other hand, when using acetone as the solvent, the selectivity changes to favor the endo-dig product. These solvent effects suggest that the 5-exo cyclization is kinetically favorable and gives the product as long as the cyclized carbanion is rapidly trapped by protonation, whereas in an aprotic solvent like acetone, the initially formed 5-exo-dig vinyl anion has sufficient time to rearrange into the more stable 6-endo-dig product.

### 3.3. Use of Strain Effects to Favor 6-endo Selectivity

**Scheme 18:**
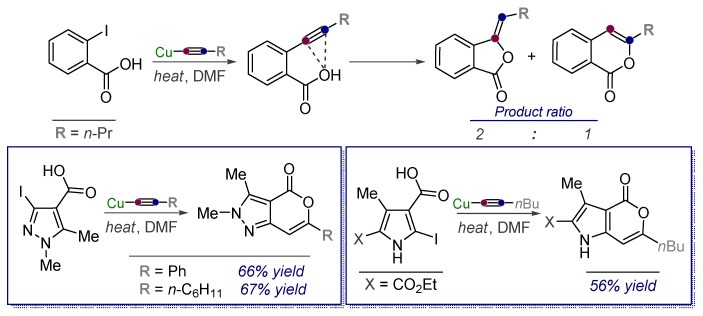
*Top:* The cyclization of o-carboxy acetylenes, formed via cuprate addition, prefers the 5-exo-dig pathway. *Bottom:* Regioselective 6-endo-dig cyclizations of acetylenic carboxylates at five-membered rings.

Castro and co-workers showed that the condensation of *o*-halobenzoic acids with substituted copper acetylides in DMF or pyridine leads, in most cases, to 5-membered lactones. Only in the case of *N*-propylacetylide has some of the 6-endo-product been observed (Scheme 18, phthalide to isocoumarin ratio 2:1) [62,63] The analogous cyclization of *O*-ethynylbenzoic acid also yielded the *γ*-lactone product of 5-exo-dig closure [64].

Interestingly, the 5-exo-dig preference observed for the benzoic acid derivatives can be completely overruled when a more strained five-membered heterocyclic core is used. A variety of *N*-containing heterocycles, shown in Scheme 18, exhibit complete 6-endo-dig selectivity [64,65,66,67]. These observations are consistent with the role of strain on the competition between closely matched radical cyclization [55,68].

### 3.4. Controlling Regioselectivity Using Elements of Thermodynamic Control

**Scheme 19:**
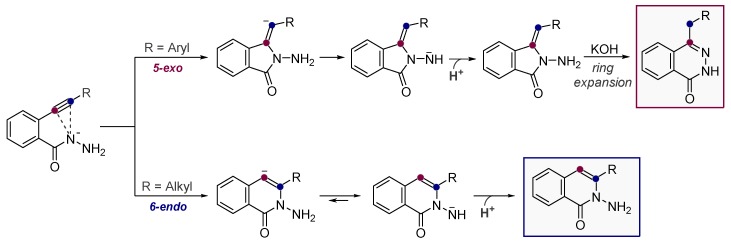
Control of *N*-nucleophilic closures by changing alkyne electronics.

Vasilevsky and Alabugin analyzed competing cyclization pathways in the cyclization of *N*-centered nucleophiles (Scheme 19) [69]. The Ph group steers the cyclization down the 5-exo path by providing benzylic stabilization to the anionic center in the product. On the other hand, the competition between the 5-exo and 6-endo-dig closures in alkyl substituted acetylenes remains tight. The activation barriers for the two cyclizations are within 1 kcal/mol from each other. However, the 5-exo-dig cyclization is predicted to be endothermic and readily reversible in solution, whereas the 6-endo-dig closure is ~10 kcal/mole exothermic.

These anionic cyclizations have a weak thermodynamic driving force because the transformation of a relatively stable nitrogen anion into a carbanionic center is inherently unfavorable. Formation of the final products is negotiated through tautomerization into a more stable anion via proton shifts. A ring expansion process is also possible for the 5-exo-products.

**Scheme 20:**
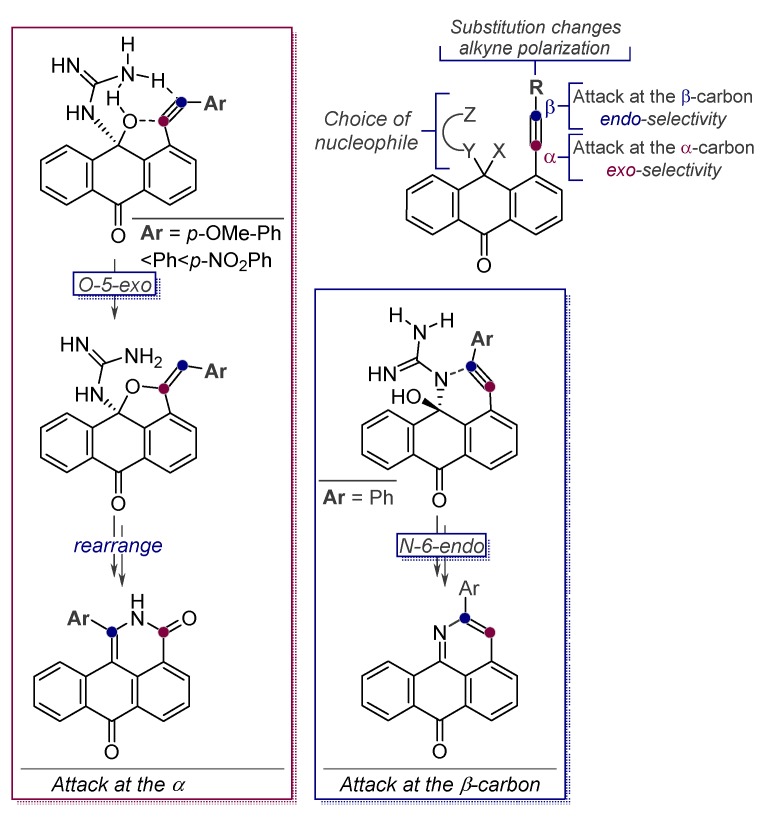
Diverging mechanistic pathways in reactions of peri-substituted acetylenyl-9,10-anthraquinones and guanidine.

Vasilevsky, Alabugin and coworkers sought a deeper understanding of the exo/endo interplay in cyclizations of alkynes by using the system shown in Scheme 20 [70]. The polyfunctional internal nucleophile corresponds a hemiaminal derived from addition of guanidine to the carbonyl moiety of peri-substituted acetylenic anthraquinones. The presence of several nucleophilic and electrophilic centers accounts for the multichannel mode of the observed reactivity. Several reaction cascades originate from alternative cyclization modes proceeding via *N*-6-endo-dig-, *N*-6-exo-dig- and *O*-5-*exo-dig* attacks. The ratio of these products is sensitive to the nature of alkyne substitution, i.e., donor Ar groups favor the formation of 6-exo-dig products [71] whereas the acceptor *p*-nitrophenyl group directs the reaction towards the heterocyclic amides via the 5-exo-dig step. The 5-exo-product undergoes a subsequent transformation which is formally equivalent to the full cleavage of the triple bond and insertion of a nitrogen atom between the two acetylenic carbons (to be discussed in Section 5).

**Scheme 21:**
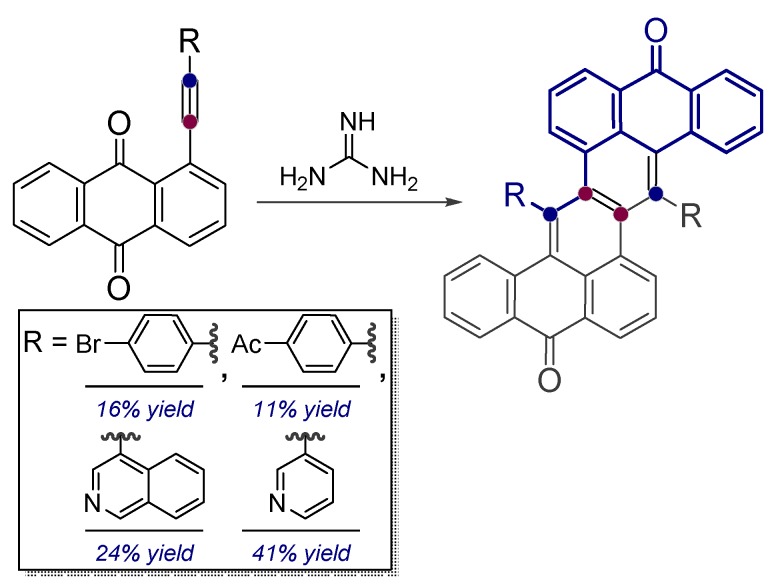
Reductive dimerization of ethynyl anthraquinones.

A particularly interesting process observed in these systems is reductive dimerization of such quinones into tetracene diones as displayed in Scheme 21 [72]. The mechanism for this transformation is still unclear.

### 3.5. Electrophile-Promoted Nucleophilic Cyclizations (EPNC)—Rendering Endo-Cyclizations Possible through “LUMO Umpolung”

**Scheme 22:**
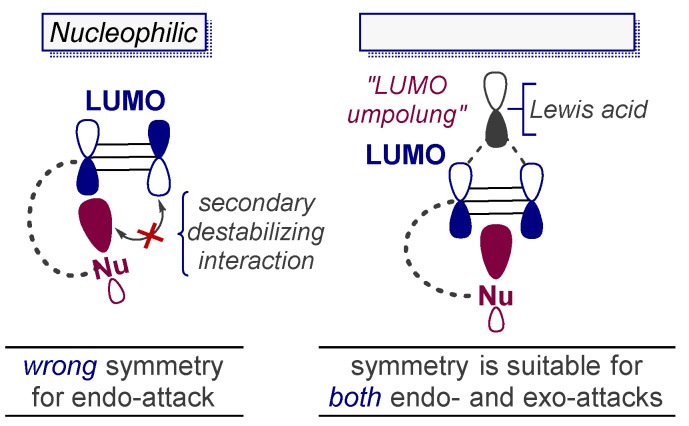
“The LUMO Umpolung”: coordination of a Lewis acid at the alkyne changes the LUMO symmetry and deactivates a destabilizing secondary orbital interaction that disfavors endo-dig cyclizations.

Coordination of an external Lewis acid to an alkyne increases alkyne electrophilicity rendering it a willing partner in many useful synthetic transformations [33]. Simultaneously, this coordination changes the nature of alkyne frontier molecular orbitals (FMOs). We named such change “LUMO Umpolung” because the LUMO of the complex resembles the HOMO of the starting alkyne [30]. This change in the orbital symmetry renders both exo-dig and endo-dig cyclizations possible in this cyclization family (Electrophile-Promoted Nucleophilic Closure (EPNC) processes; Scheme 22) [73].

**Scheme 23:**
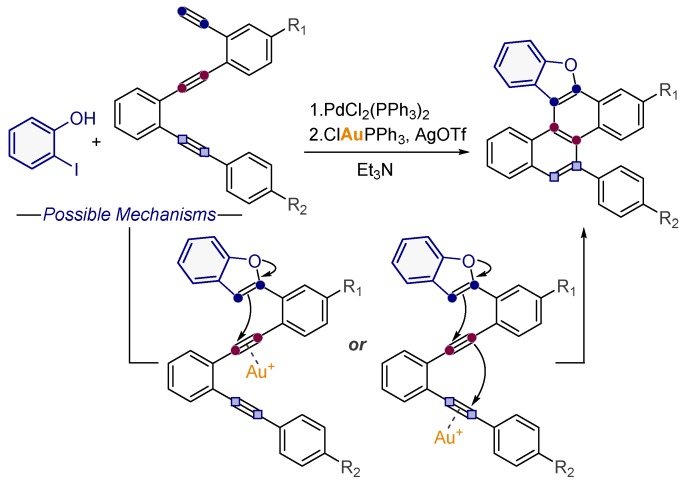
The formal “all-endo” metal-assisted cyclization cascade is initiated by a 5-endo-dig closure followed by two 6-endo-dig closures.

Once formed, the vinyl ethers can be used for the subsequent C-C bond formations if the substrate has additional appropriately activated electrophilic units. In particular, this cyclization mode has been shown to be effective in initiating an all endo-selective cascade, which fuses a polycyclic aromatic backbone to the electron-rich furan subunit [74,75,76], as shown in Scheme 23. The mechanism of the 2nd and 3rd ring formation is so far unclear and may either include sequential activation of the two alkynes as electrophilic Au-complexes or an Au-catalyzed Bergman cyclization [77] followed by coupling of the benzofuran and dehydronapthalene subunits.

### 3.6. Endo-Cascade through Vinylidene Intermediates

**Scheme 24:**
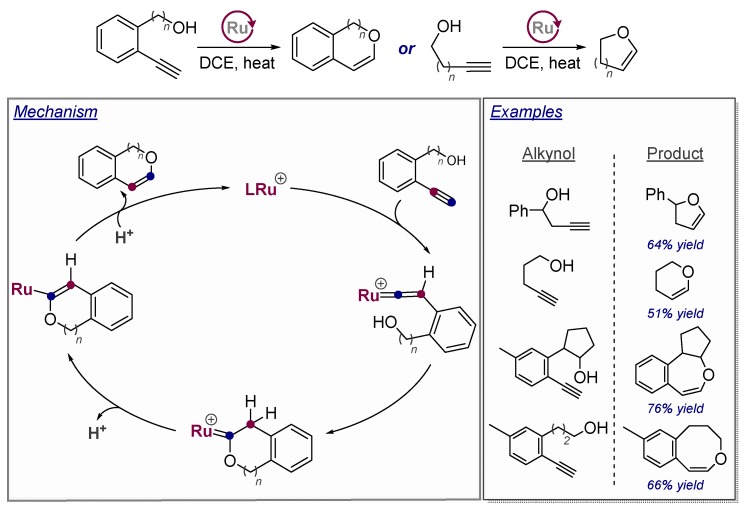
Cycloisomerizations of terminal alkynols under Ru-catalysis.

Cai et al. illustrated that the cyclization selectivity can be shifted reliably in favor of the endo-dig cyclizations by changing the reaction mechanism [78]. The 5, 6, 7, and 8-endo-dig cycloisomerizations of terminal alkynols are possible under Ru-catalysis due to the formation and trapping of the Ru-vinylidene intermediate. Through this robust protocol, the preparation of “non-branched” cyclic ethers of different size is achievable in a modular and efficient way (Scheme 24).

The next section shows how enol ethers prepared from alkynes can be involved into additional in situ transformations that add synthetic value to the product.

## 4. Petasis-Ferrier Rearrangement

**Scheme 25:**
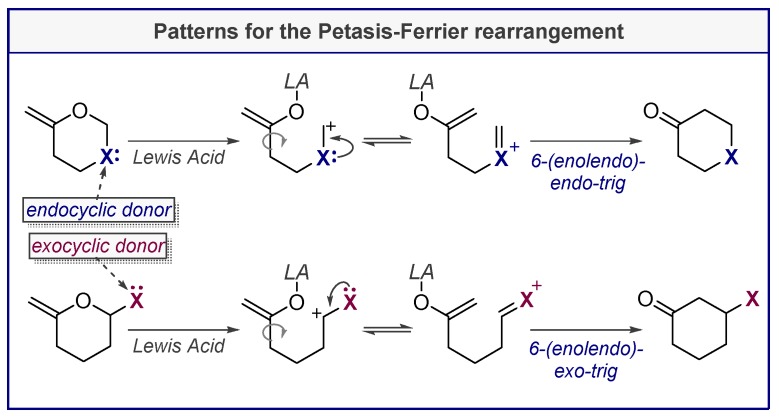
Two possible stabilization patterns for the Petasis-Ferrier rearrangement.

The connection between alkynes and carbonyls is also illustrated by Petasis-Ferrier rearrangement (PFR). PFR is a valuable process that utilizes the dual (O- vs. C-) reactivity of enolates for the controlled formation of C-C bonds via an isomerization [79,80,81,82,83,84]. In classic version of the PFR, a cyclic vinyl ethers undergoes an acid-catalyzed ring opening via the C-O bond scission (the reverse of enol *O*-cyclization with a cationic electrophile). Such scission forms an enol, concomitantly with a cationic center. The latter serves as an electrophile that can, in the last step of the PFR cascade, recapture the enol at the *C*-terminus of the latter. This C-C bond formation via a recyclization step furnishes the new cyclic product Scheme 25. The cationic intermediate usually has to be stabilized (typically by a lone pair of an adjacent heteroatom) in order for the C-O scission process to be thermodynamically feasible. The Au-catalyzed versions of PFR utilize homopropargylic derivatives as carbonyl precursors [85,86,87,88,89,90,91,92].

**Scheme 26:**
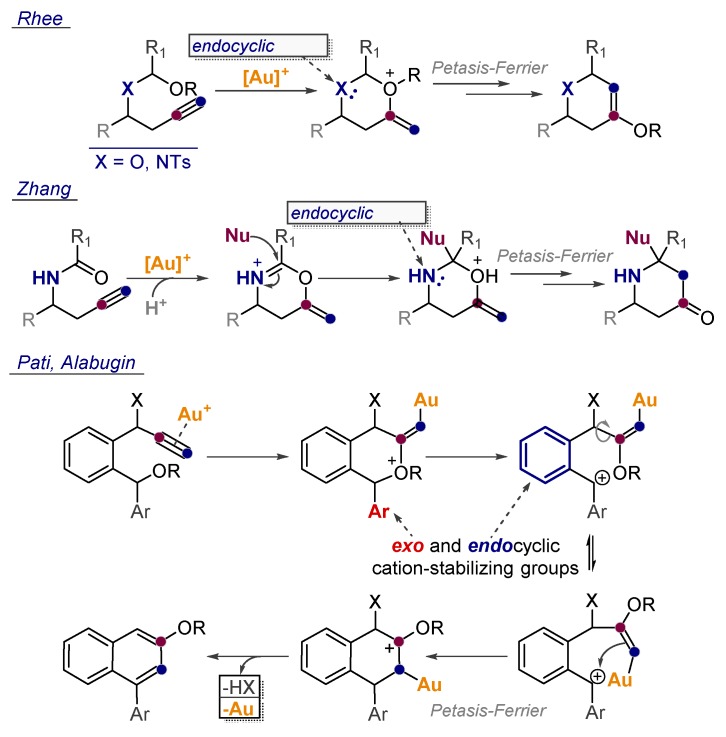
Au-catalyzed versions of the Petasis-Ferrier reaction. Top: cation stabilization by an endocyclic donor assists transformation of homopropargylic esters and amides into heterocyclic products. Bottom: cation stabilization by an exocyclic donor assists transformation of *ortho*-alkynyl benzyl methyl ethers into naphthalenes.

The Au-catalyzed PFR cascades developed by the groups of Rhee [85,86] and Zhang [87,88] are initiated as the enol ether formed via an *O*-nucleophilic attack at an activated alkyne/Au(I) π-complex (Scheme 26, top). The fragmentation of the vinyl ether intermediate is typically assisted by the presence of an endocyclic heteroatom donor. Such reactions were successfully used for the preparation of biologically active heterocycles. Subsequently, Pati and Alabugin reported that, when an aromatic ring can be used as an endocyclic donor with the assistance of a properly positioned exocyclic aryl donor, the Au-catalyzed PFR can be utilized for synthesis of carbocyclic aromatic systems (Scheme 26, bottom) [93].

Overall, the mild and efficient Au-catalyzed Petasis-Ferrier/aromatization sequence converts alkynes into activated enol ethers. Alkoxy substitution in the products can be used for subsequent synthetic transformations, such as the highly regioselective oxidative dimerization into tetranaphthyls [93].

## 5. The “Oxidant-Free Nitrogen Baeyer-Villiger Rearrangement”

In this section, we will show a cascade transformation of an alkyne that involves two “carbonyl reactions”, the Petasis-Ferrier reaction and an “aza Baeyer-Villiger (BV) rearrangement”. The Baeyer-Villiger reaction provides an important synthetic connection between ketones and esters [94,95,96,97,98,99]. More than a century after its discovery, this transformation still continues to provide a valuable connection between these key organic functional groups.

**Scheme 27:**
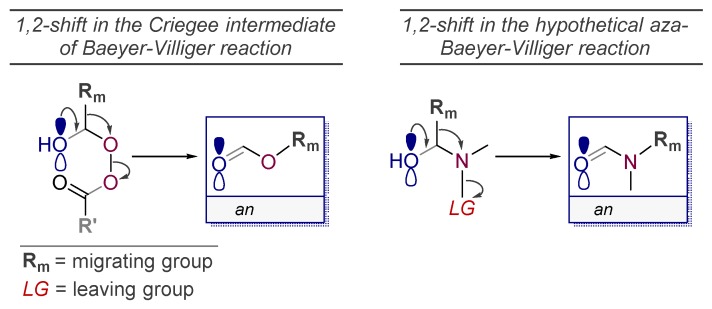
1,2-shifts in the Baeyer-Villiger and aza-Baeyer-Villiger reactions.

The key step in the mechanism of Baeyer-Villiger (BV) rearrangement involves a 1,2-alkyl shift in a tetrahedral intermediate formed by the addition of a peroxyacid to the carbonyl group of an ester (i.e., the Criegee intermediate) [100]. This reaction is assisted, by exchange of a weak O-O bond [101] into a more stable C-O bond and two stereoelectronic effects [102,103,104,105]. The key participants include the *p*-type lone pair of O_1_, the breaking C_2_-R_m_ bond and the O_3_-O_4_ acceptor (Scheme 27). The “primary stereoelectronic effect” requires antiperiplanarity of the breaking O-O bond and the migrating C-R_m_ bond. The “secondary effect” is switched on when the lone pair of the O_1_H group aligns with the breaking C-R_m_ bond [106,107].

**Scheme 28:**
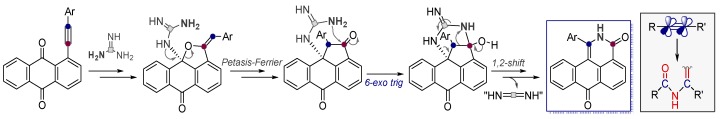
Alkyne “disassembly” via carbonyl cascades leading to nitrogen insertion between alkyne carbons. Note that the fragmentation−recyclization sequence is analogous to the Petasis−Ferrier rearrangement whereas the [1,2]-shift can be considered as an aza-analogue of the Baeyer-Villiger reaction.

An aza-version of the BV reaction would open a direct synthetic path from ketones to amides. This process would require the 1,2-shift to break the N-X bond between nitrogen and a leaving group X. As far as we know, there is no example of such reaction that starts directly from a carbonyl precursor and proceeds via a hemiaminal intermediate [108]. However, use of an alkyne starting material led to the discovery of an aza-BV reaction in a cascade where this process is coupled with a Petasis-Ferrier reaction [70,72,109,110]. The outcome of this cascade is quite remarkable—this sequence of reactions inserts a nitrogen atom between the two alkyne carbons (Scheme 28). The overall transformation leads to the formation of *six* new bonds at the two alkyne carbons with complete disassembly of the alkyne moiety. Note that this cascade alkyne transformation is mediated by classic carbonyl chemistry, i.e., the fragmentation−recyclization sequence is similar the Petasis−Ferrier rearrangement and the [1,2]-shift converting the cyclic heminal into lactam is the aza-analogue of the Baeyer-Villiger oxidation (except that it is done without any oxidants!).

Like the BV, the “aza-BV” involves a 1,2-carbon shift at the carbon atom substituted with two heteroatoms (hemiaminal rather than hemiacetal). In both processes, the C-C bond scission is assisted by the C=O bond formation and scission of a bond at the heteroatom that accepts the migrating group. In the classic BV, the breaking bond is the O-O bond whereas in the present version of the aza-BV, the breaking bond is the N-LG bond, where LG is a C-centered leaving group.

## 6. Alkynes as Carbonyl Surrogates in the Synthesis of Aldol Products

In the previous sections, we have shown the utility of alkynes in “heterocyclizations” via reactions with heteroatomic nucleophiles. In this section, we will illustrate the value of alkynes for the CC bond formation in “carbocyclizations”. The aldol condensation is one of the classic synthetic transformations that takes advantage of the dual ability of the carbonyl functionality to serve as a source of both the electrophile (carbonyl) and nucleophile (enol/enolate) partners for the C-C bond formation. However, these transformations often depend on chemo-selective formation of an enolate from one carbonyl precursor in the presence of another [111,112]. The challenge arises mainly because of the different possible enolate formations, an issue that is inherent to the aldol condensation, with symmetric ketones being the exception. Exploiting alkynes as hidden carbonyl precursors allows for the use of each of the alkyne carbons in each of the alkyne units as either a nucleophilic or an electrophilic partner depending on the activation method (Scheme 4).

**Scheme 29:**
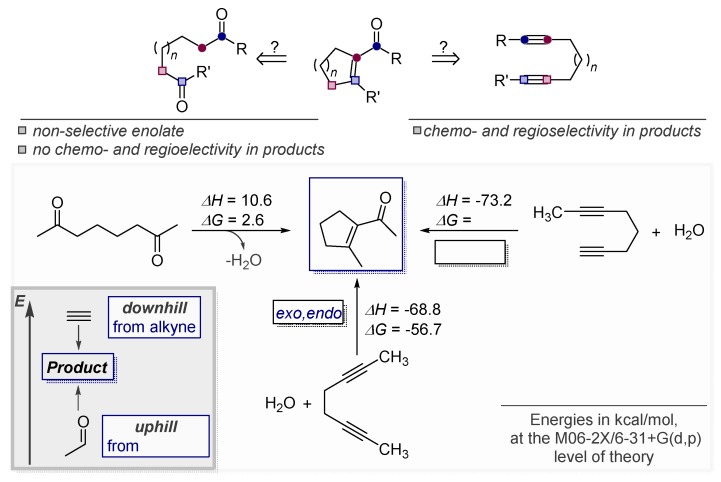
Synthesis of cyclic enones from dicarbonyls and diynes.

To understand the advantages of alkyne as a better carbonyl surrogate, we have compared thermodynamics for the synthesis of cyclic enones as depicted in Scheme 29. The calculations shows that transformations of both model alkynes octa-2,6-diyne and octa-1,6-diyne into 1-(2-methyl-cyclopent-1-en-1-yl)ethanone are highly exergonic (−56.7 Kcal/mole and −59.7 kcal/mole, respectively). However, accessing the same enone from the carbonyl precursor, octane-2,7-dione, is uphill by 2.6 kcal/mole. These comparisons clearly illustrate that one can utilize alkynes stored energy efficiently in the synthesis of aldol products.

For the preparation of non-symmetric enones, one has to match precisely the electrophilic and nucleophilic components by the design of starting materials. In alkynes, this match can be done via catalyst design, so each of alkynes can be either the E^+^ or Nu^−^ partner.

**Scheme 30:**
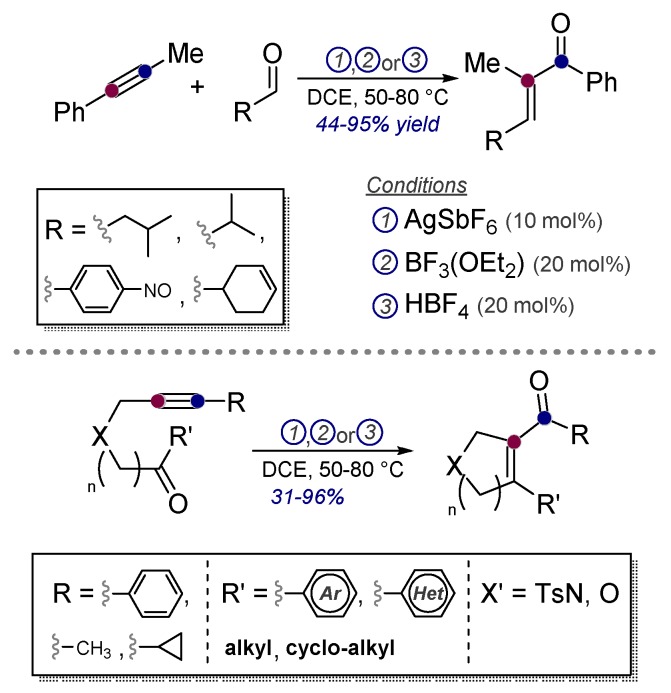
Use of alkynes as enolate equivalents in the formal aldol condensations with aldehydes (“alkyne-carbonyl metathesis”).

Acid-catalyzed reactions of alkynes and aldehydes allow to use alkynes as enolate equivalents in aldol condensations (Scheme 30).

**Scheme 31:**
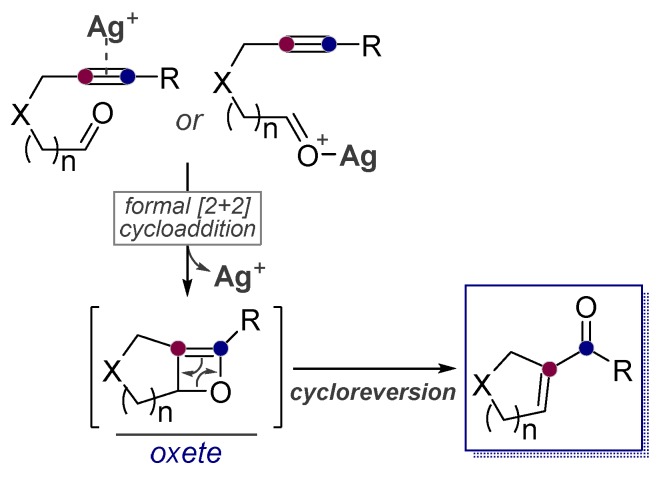
Suggested mechanism of the alkyne-carbonyl “aldol condensations”.

Several intra- and intermolecular versions of this alkyne-aldehyde coupling successfully provided trisubstituted enones in the presence of Lewis and Brønsted acids (AgSbF_6_, BF_3_(OEt_2_), or HBF_4_) [113]. This reaction proceeds in higher yields under the catalytic activation by AgSbF_6_. Intermolecular coupling proceeds stereoselectively with the formation of a single geometrical isomer. The suggested mechanism of the alkyne-carbonyl reaction involves complexation of Ag(I) with either the alkyne or the carbonyl oxygen that facilitates a formal “2 + 2” cycloaddition that leads to the formation of unstable oxete intermediate. As shown in Scheme 31, This strained heterocycle can undergo a cycloreversion to conjugated enone.

Subsequent research greatly expanded synthetic utility of these reactions to include ketones and non-activated alkynes [114,115,116,117]. Considering the mechanism, one can classify this process as a formal alkyne-carbonyl “metathesis” where the carbonyl double bond is “transferred” to one of the alkyne carbons [118,119,120,121]. This process was suggested to serve as “a completely atom economical alternative to the use of stabilized Wittig reagents in carbonyl olefination” [113]. One can also consider this process as another example where alkyne behaves as a 1,2-dicarbene equivalent.

**Scheme 32:**
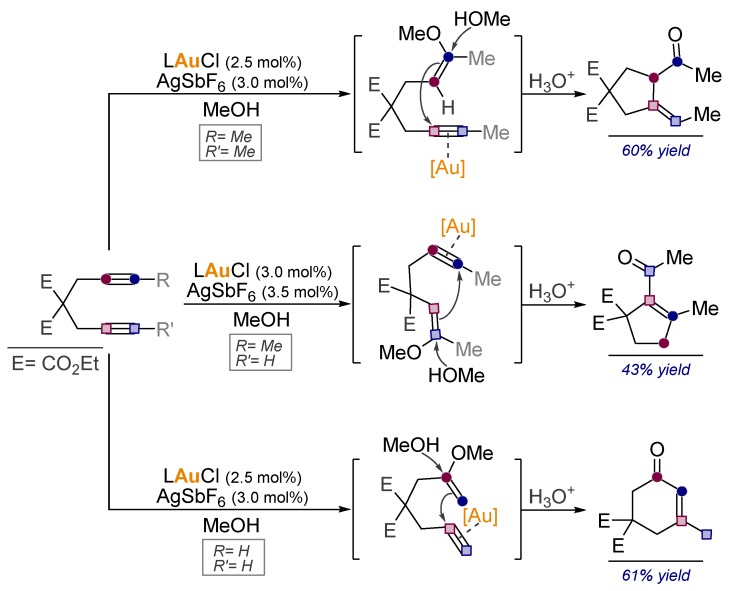
Au-catalyzed hydrative cyclizations of diynes.

Due to the alkyne/carbonyl equivalency, one can initiate similar intramolecular cascades by starting from a diyne rather than a ynone or an ynal. An example of this chemistry is provided by Au-catalyzed hydrative cyclizations of diynes (Scheme 32) [122].

The proposed mechanism for this transformation includes transformation of one of the alkynes into an enol ether (the nucleophilic component) and conversion of the other alkyne into an electrophile via a π-complex with the catalyst. After cyclization, the resulting ketal is transformed into the corresponding ketone by hydrolysis.

**Scheme 33:**
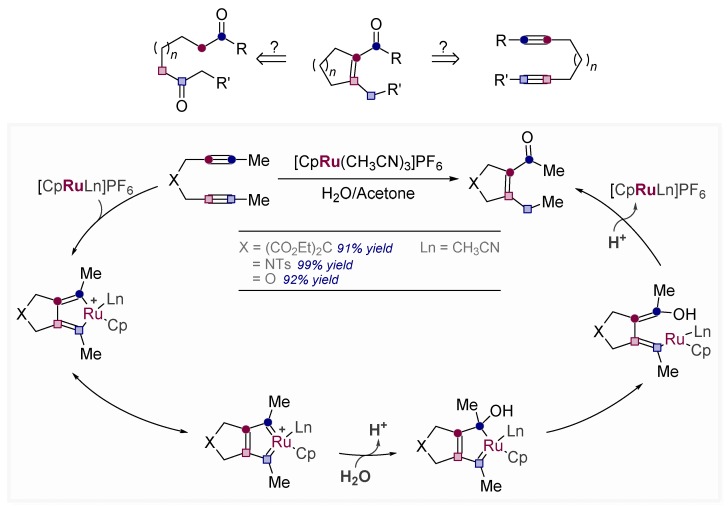
Ruthenium catalyzed hydrative cyclization of diynes.

Furthermore, one can make the enone products of formal aldol condensations from alkynes by bypassing the polar path for the CC bond formation altogether. Trost and coworkers illustrated this possibility in ruthenium-catalyzed synthesis of cyclic enones from diyne precursors (Scheme 33). In this process, water was used as the reagent for unmasking the hidden carbonyl after the CC bond formation [123]. The initial step involves formation of “metallacyclopentadiene” intermediate which then undergoes regioselective H_2_O attack at less hindered site giving desired product. A complete chemoselectivity was observed for significantly bulkier groups affording single regioisomer.

In this reaction the use of Ru-catalyst allows one to use the dicarbene character of alkynes. In the initial stage, the C=C moiety is formed by coupling the internal alkyne carbons and leaving the external carbons as two metal carbenoids (show this conceptually). The difference of this design is that the alkyne-carbonyl conversion is done at the final step of reaction (rather than by nucleophilic attack at the alkyne) by the hydration step that releases the Ru-catalyst and furnishes the C(O) and CH_2_ groups from the two C=Ru moieties.

### Alkynes in Retro-Aldol and Retro-Mannich Fragmentations

**Scheme 34:**
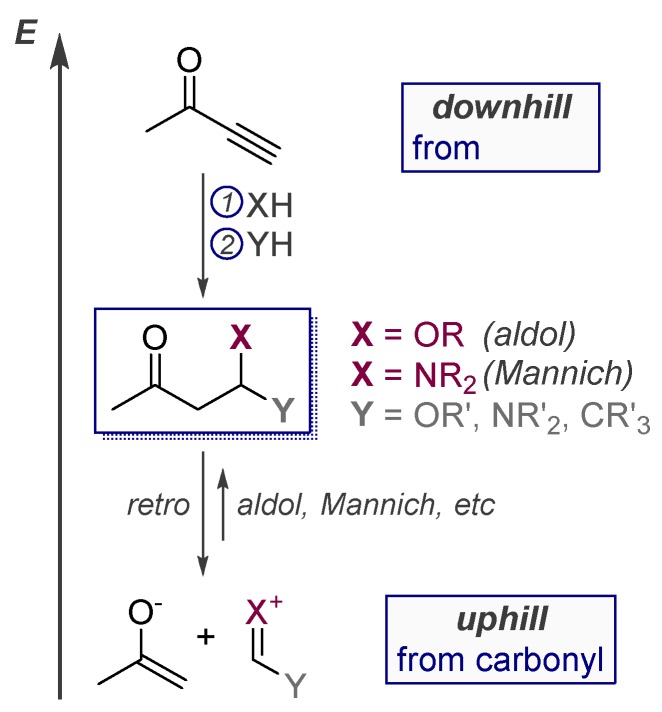
Use of alkyne high energy and cross-over to the “carbonyl reaction field” for the full disassembly of triple bond.

As alkynes are higher in energy than carbonyls, alkynes can be converted back to carbonyls via addition/isomerization (Scheme 34). In addition to the simple hydrolysis, more interesting transformations involve conversion of substituted alkynes to the products of reactions that would be endergonic if started from a carbonyl precursor. For example, in those cases where aldol or similar condensation/addition product is less stable than its carbonyl precursors, accessing such products from the alkyne side leads to their further reaction via a retro-addition (i.e., fragmentation) mode.

**Scheme 35:**
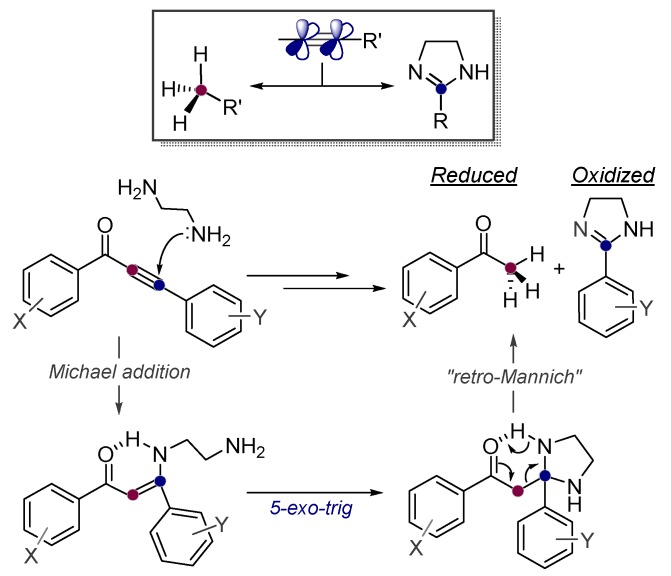
Complete scission of the triple bond in keto alkynes mediated by the retro-Mannich reaction.

Recently, several examples of such reactions which proceed via the retro-Mannich route were described by Vasilevsky, Alabugin and coworkers [124]. These reactions can be used for cleaving the triple bonds under relatively mild conditions. For example, the full alkyne fragmentation can be induced in the reaction of ethylene diamine with diarylketoacetylenes. This process leads to the formation of acetophenones and 2-substituted imidazolines (Scheme 35).

In these transformations, controlling the regiochemistry of the ketone formation is crucial for the success. Alkynes with acceptor substituents undergo Michael addition producing a relatively stable enamino ketone that can be isolated. However, at higher temperatures this intermediate undergoes a stereoelectronically favorable [49,125] 5-exo-trig cyclization followed by efficient retro-Mannich fragmentation. The fragmentation is assisted by a stereoelectronically optimal interaction [126] of the breaking C-C bond with lone pair of one of the nitrogen atoms.

**Scheme 36:**
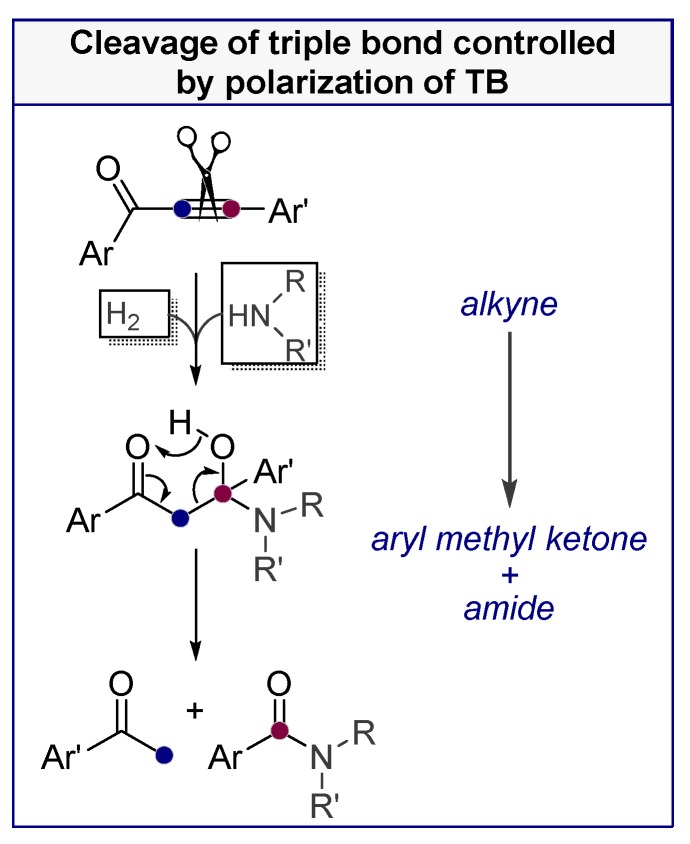
Variations of retro-Mannich-mediated alkyne fragmentations with the 2nd nucleophilic attack being *inter*molecular.

It is important to note that in order to keep the overall reaction as a “redox neutral” process, the final fate of the two alkyne carbons has to be opposite. While one of the carbons ends as part a methyl group (reduced relative to alkyne), the other one ends up as C1 of a dihydroimidazole (oxidized relative to alkyne; Scheme 36).

**Scheme 37:**
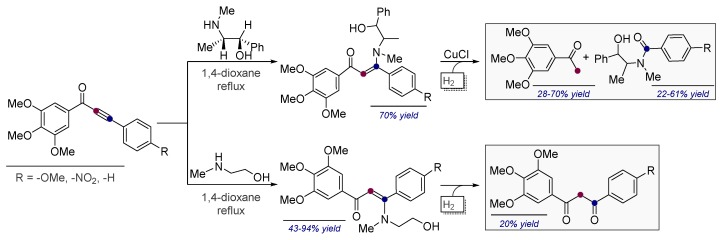
Reaction of *α*-alkynylketones with aminoalcohols.

Reactions of *α*-alkynylketones with other type of binucleophiles, aminoalcohols, proceed in a more complex manner [127]. The effects of electronic and steric factors were investigated by choosing substrates containing donor (*p*-methoxyphenyl) and acceptor (phenyl and *p*-nitrophenyl) substituents. The choice of nucleophiles was expanded to include 2-aminoethanol, 2-(methyl-amino)ethanol and the more sterically hindered 2-(methylamino)-1-phenylpropan-1-ol. Intermolecular addition to α-ketoacetylenes lead to corresponding enamines (Scheme 37, bottom).

The subsequent intramolecular Michael addition step is sensitive to the nature of the amine. The introduction of additional methyl and phenyl substituents in ethanolamine dramatically changes the direction of the process. In the reaction of α-ketoacetylenes with more sterically hindered partners, the transformation of the initial adduct to the products of the full triple bond cleavage required more stringent conditions (Scheme 37, top).

**Scheme 38:**
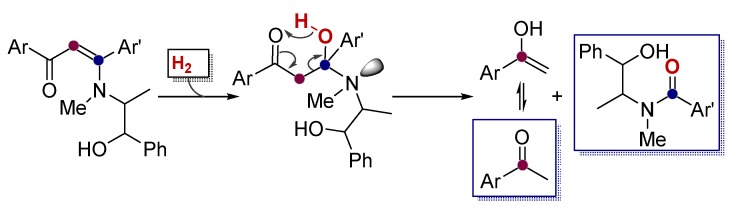
Reaction of α-ketoacetylenes with pseudoephedrine.

The suggested pathway of this cascade includes addition of a water molecule to the enamine and then, through 6-membered transition state (TS) and by stereoelectronic assistance of the exocyclic nitrogen leading to the final C-C cleavage -formation of amides and the common methyl aryl ketones. The fragmentation step is likely to be assisted by intermolecular proton transfer to the developing negative charge at the carbonyl oxygen from the properly positioned N-H bond within the six-membered transition state TS (Scheme 38).

**Scheme 39:**
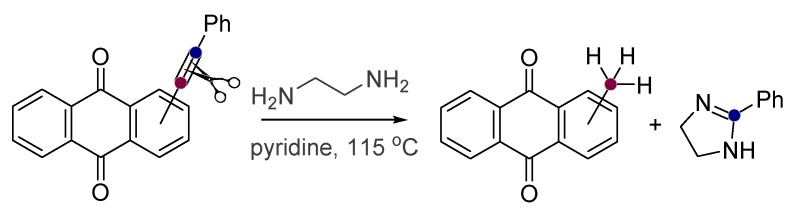
C≡C bond scission in 1- and 2-phenylethynyl-9,10-anthraquinones.

Both 1- and 2-phenylethynyl-9,10-anthraquinones, the vinylogs of α-ketoacetylenes in which the carbonyl group is removed further away from the triple bond can also be involved into full scission of the C≡C bond. However, conditions are harsher than in the case of α-acetylenic ketones. The fragmentation proceeds most effectively in refluxing pyridine with the 50-fold excess of the nucleophile (Scheme 39) [128].

**Scheme 40:**
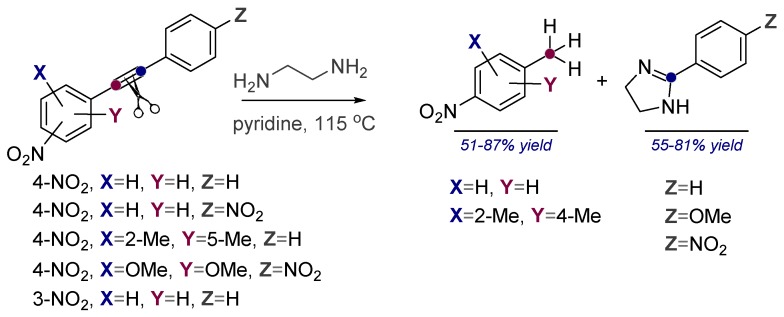
Expanded alkyne fragmentation reactions to compounds containing varied functionalities.

More important is the possibility of expanding the alkyne fragmentation reactions to compounds with other functional groups, also positioned away from the alkyne moiety by the example of family of nitro-substituted diaryl alkynes with different positions of the acceptor NO_2_-group (Scheme 40) [129].

Thus, presence of a *para* nitro group is sufficient for the triple bond scission in the reaction with 1,2-diaminoethane, leading to the fragmentation products: 1-methyl-4-nitrobenzene (68%) and 2-phenylimidazoline (61%). Electron-deficiency of the alkyne moiety is more important than alkyne polarization. For example, 1,2-bis(4-nitrophenyl)ethyne, a symmetric alkyne with two acceptor groups, reacts faster (1 h), yielding 51% *p*-nitrotoluene and 55% imidazoline.

**Scheme 41:**
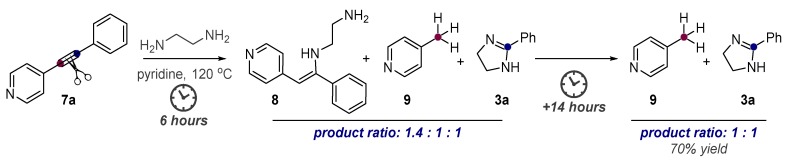
Alkyne fragmentation reactions in pyridine containing substrates.

This chemistry can be expanded to alkynes with electron-deficient heterocycle substituents such as the pyridine moiety (Scheme 41). For example, 4-pyridinyl alkyne was fully transformed in the two fragmentation products, 2-phenyl-4,5-dihydroimidazole and 4-methylpyridine (picoline).

In summary, reaction of electron-deficient alkynes with ethylene diamine is a general transformation that can involve not only α-acetylenic ketones but other suitably activated alkynes.

**Scheme 42:**
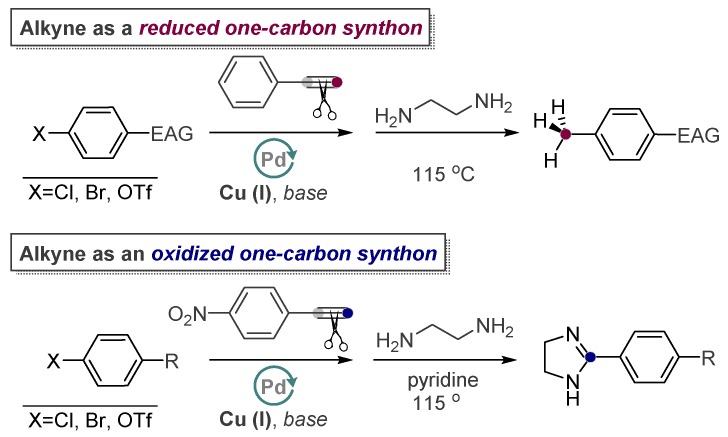
Retrosynthetic equivalency of alkynes and methyl group (top) and protected carboxylic acids (bottom).

From a synthetic perspective, the combination of Sonogashira cross-coupling and fragmentation presented in this work opens the door for two potentially useful synthetic transformations: introduction of methyl groups to electron-deficient aryl halides or triflates or introduction of masked carboxyl (imidazoline) into donor aryl halides or triflates. These transformations suggest retrosynthetic equivalency of alkynes and one-carbon synthons, in the most reduced and oxidized forms of the latter (Scheme 42).

**Scheme 43:**
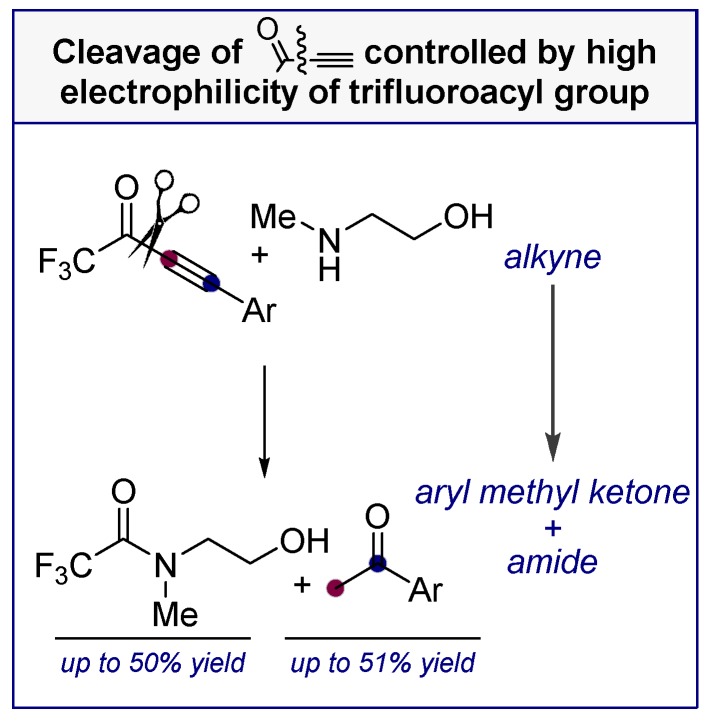
Reaction of CF_3_-ynones with amino alcohols.

It is also interesting that reaction of the *β*-ethanolamines with CF_3_-ynones follows a different path where the C(sp^3^)-C(sp) bond of the enone is broken (Scheme 43) [129,130].

This interesting example demonstrates the role of the nature of the acyl group in the direction of the aza-Michael-addition of β-aminoethanols to α-alkynyl ketones. Reaction of CF_3_-ynones with amino alcohols transform ketoacetylenes into two carbonyl compounds -trifluoroacetylated amides and methylarylketones.

## 7. Alkynes in the Synthesis of α-oxo Gold Carbenes

**Scheme 44:**
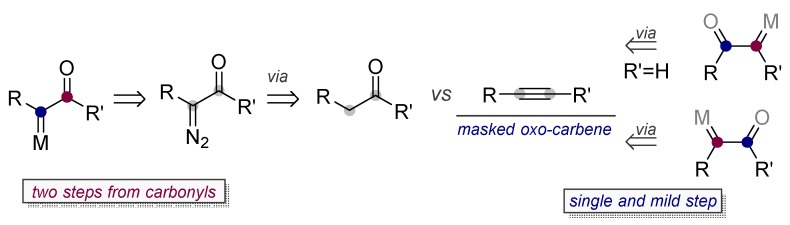
Retrosynthetic analysis of two potential routes to metal-carbenes from alkynes and ketones.

In this section, we give an example of an oxidative alkyne transformation that leads to a carbonyl derivative with an additional useful functionality. The versatility of metal carbenes makes them useful intermediates for synthetically challenging transformations. A common approach to alpha-oxo metal carbenes is the metal catalyzed decomposition of diazo-carbonyl compounds that can be synthesized from carbonyl precursors containing an active alpha methylene group [131,132]. Despite its synthetic utility, these highly energetic diazo-intermediates are potentially explosive and hazardous. Under the right conditions, alkynes allow for another approach towards α-oxo carbenoids that obviates the need of diazo-derivatives (Scheme 44). This transformation is not surprising since, as mentioned in previous sections, alkynes can be considered as super-stabilized 1,2-dicarbenes. In the presence of a suitable oxidant and a catalyst their oxidation yields α-oxo-gold carbenes in an operationally easy and environmentally benign fashion [133,134]. Both intermolecular and intramolecular oxidation methods of alkyne for transformation to oxo-gold carbene have been developed [135].

**Scheme 45:**
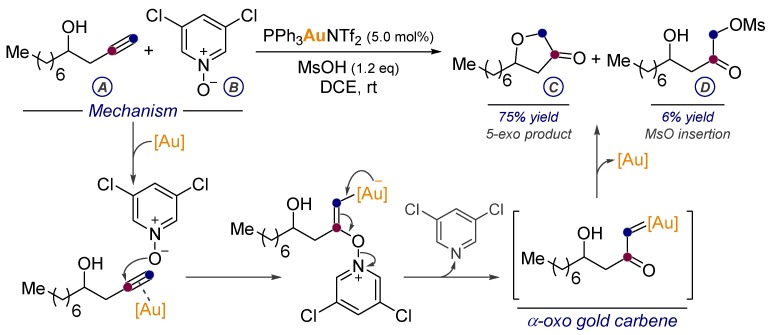
α-Oxo gold carbenes via Au-catalyzed alkyne oxidation.

Among many reports, Zhang and coworkers reported one of the early examples of accessing gold carbenes from alkynes using an external organic oxidant [136]. In particular, a mild approach to α-oxo carbenes involves Au-catalyzed intermolecular oxidation of terminal alkynes where pyridine *N*-oxides act as the oxidant (Scheme 45). It was proposed that after the initial addition of *O*-nucleophile to Au-activated alkyne of homopropargyl alcohol, expulsion of pyridine and back donation from gold [137] yields the desired α-oxo gold carbene intermediates. The Au-carbene can then undergo an insertion reaction with the pendant OH group, yielding dihydrofuran-3-ones in good yields. Mesyl alcohol is used as an additive in order to remove the basic pyridine side product that could poison the Au-catalyst. Formation of α-oxo gold carbene was further supported by isolation of mesylate as an insertion product.

## 8. Alkynes as Carbonyls in the Rautenstrauch Rearrangement

**Scheme 46:**
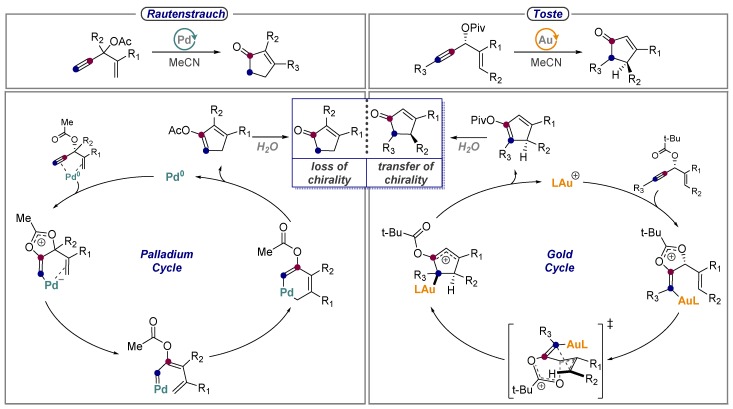
Palladium and Gold catalyzed cycloisomerizations of 1-ethynyl-2-propenyl acetates to 2-cyclopentenones.

An example that showcases how the utilization of alkynes can expand well established carbonyl chemistry (e.g., the Nazarov reaction) is the Rautenstrauch rearrangement. The Rautenstrauch rearrangement, a Pd-catalyzed variant of the Nazarov cyclization [138] was initially reported as a way to facilitate the transformation of 1-ethynyl-2-propenyl acetates to the corresponding 2-cyclopentenones (Scheme 46, left) [139]. Of crucial importance for this transformation is the presence of the acetate group in the starting enyne. First, it is the synergy between the roles of the acetate group and the alkyne moiety that allows for the “unmasking” of the alkyne as a carbonyl equivalent -for this unmasking to happen, the acetate has to undergo a 1,2 migration from its original position (the propargyl position) forming a cyclic intermediate. The latter is believed to give access to palladacarbene species from which a Nazarov-type cyclization onto the metal center is possible.

Although the Ratenstrauch rearrangement offers an efficient route to disubstituted cyclopentenones, its biggest limitation is that only achiral cyclopentenones substituted at the 2 and 3 positions can be prepared through this method. The loss of chiral information from optically active starting materials has been proposed as experimental evidence that the metallocarbene-intermediate mechanism is operational [140,141,142,143,144]. These limitations were later overcome by the Toste group who developed an Au-catalyzed variant of the Rautenstrauch reaction. In this transformation, the substitution pattern of the accessible cyclopentanone products was expanded to the 3, 4, and 5 positions [145]. Furthermore, their approach allowed for the transfer of chiral information from the starting eneyne to the cyclopentanone (Scheme 46, right). This interesting result gave suggested that, under the gold catalysis, a different mechanism bypassing the metallocarbene may be operational. In order to explain the reaction’s stereoselectivity, it was proposed that C-C bond formation needs to happen prior to C-O scission on the stereogenic center, and that the transformation could pass through a transition state where the breaking C-O bond is orthogonal to the plane of the olefin during the Nazarov-type step.

## 9. Converting Alkynes to Carbonyls Via Pericyclic Reactions

Anionic oxy-Cope rearrangement of bis-alkynes produced by reaction of acetylides with benzil proceeds below room temperature and continues via electrocyclic ring closure [146]. Computational studies confirm significant barrier decrease for the rearrangement where the central C-C bond is weakened by the oxyanionic radical stabilizing groups. This cascade offers another entry point into carbonyl chemistry from bis-propargylic bis-alkynes.

**Scheme 47:**
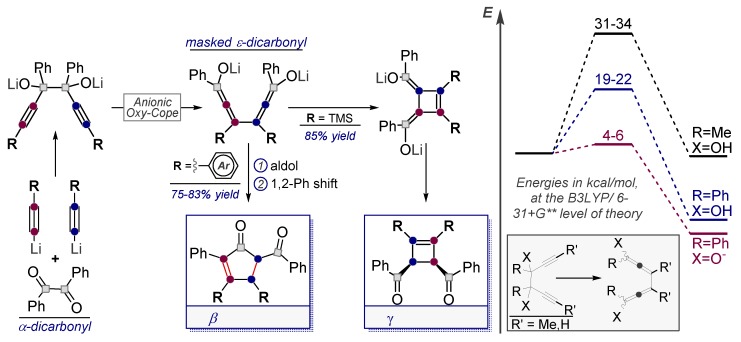
Insertion of cyclobutene ring between two carbonyl carbons via pericyclic chemistry of alkynes.

Because of the presence of two hydroxyl groups at the central bond of the bis-acetylenes, these compounds can be considered as a latent dicarbonyl functionality that is revealed by the oxy-Cope process in its bis-enolized state. As a result, one can couple the pericyclic step with typical carbonyl chemistry, such as intramolecular aldol condensations (Scheme 47).

## 10. Conclusions

With broad strokes, we have illustrated a few opportunities that arise from the synthetic equivalency of carbonyl compounds and alkynes. Considering the large body of the published work, the overview is certainly not comprehensive, but we hope that it will serve as a helpful reminder of this useful connection between two of the most common and important functional groups of organic chemistry. Such connections should not be overlooked in the search for efficient synthetic strategies.
